# Strain distribution of repaired articular cartilage defects by tissue engineering under compression loading

**DOI:** 10.1186/s13018-018-0726-0

**Published:** 2018-01-30

**Authors:** Shilei Wang, Yan Bao, Yinjie Guan, Chunqiu Zhang, Haiying Liu, Xu Yang, Lilan Gao, Tongtong Guo, Qian Chen

**Affiliations:** 1 Tianjin Key Laboratory of the Design and Intelligent Control of the Advanced Mechatronical System, Tianjin University of Technology, Tianjin, 300384 China; 2 Cell and Molecular Biology Laboratory, Department of Orthopaedics, Alpert Medical School of Brown University/Rhode Island Hospital, 1 Hoppin St., Ste. 402, Providence, RI 02903 USA; 3 Nature Science Department, Harbin Institute of Technology, Shenzhen Campus, Shenzhen, 518055 China

**Keywords:** Cartilage tissue engineering, Cartilage defects, Strain distribution, Digital image correlation

## Abstract

**Background:**

It is difficult to repair cartilage damage when cartilage undergoes trauma or degeneration. Cartilage tissue engineering is an ideal treatment method to repair cartilage defects, but at present, there are still some uncertainties to be researched in cartilage tissue engineering including the mechanical properties of the repaired region.

**Methods:**

In this study, using an agarose gel as artificial cartilage implanted into the cartilage defect and gluing the agarose gel to cartilage by using the medical bio-adhesive, the full-thickness and half-thickness defects models of articular cartilage in vitro repaired by tissue engineering were constructed. Strain behaviors of the repaired region were analyzed by the digital correlation technology under 5, 10, 15, and 20% compressive load.

**Results:**

The axial normal strain (E*x*) perpendicular to the surface of the cartilage and lateral normal strain (E*y*) as well as shear strain (E*xy*) appeared obviously heterogeneous in the repaired region. In the full-defect model, E*x* showed depth-dependent strain profiles where maximum E*x* occurs at the low middle zone while in the half-defect mode, E*x* showed heterogeneous strain profiles where maximum E*x* occurs at the near deep zone. E*y* and E*xy* at the interface site of both models present significantly differed from the host cartilage site. E*y* and E*xy* exhibited region-specific change at the host, interface, and artificial cartilage sites in the superficial, middle, and deep zones due to the artificial cartilage implantation.

**Conclusion:**

Both defect models of cartilage exhibited a heterogeneous strain field due to the engineered cartilage tissue implant. The abnormal strain field can cause the cells within the repaired area to enter complex mechanical states which will affect the restoration of cartilage defects.

## Background

Articular cartilage repair and regeneration continue to be largely intractable due to its poor regenerative properties [1]. Traditional methods like autografts, allografts, or microfracture have been clinically employed to treat articular cartilage defects, but there are still many shortcomings associated with these therapies [2]. Articular cartilage tissue engineering holds great promise to repair, regenerate, and/or improve injured or diseased articular cartilage functionality without the shortcomings.

In the past decades, enormous progress has been made in the optimization of strategies for cartilage tissue engineering. Cartilage tissue engineering has been used in clinical treatment, but to date, there are still many issues that need to be researched [3, 4]. In fact, after construct implantation, new tissue and surrounding host cartilage may degenerate [5]. Cartilage repair often results in fibrocartilaginous tissue without the production of normal hyaline cartilage [4, 6]. The integration between the host cartilage and repair tissue is not successful enough [7]. The exact mechanism of progressive degeneration has not been elucidated due to many causes, but from the aspect of cartilage mechanobiology, a mechanical role can be suggested in this process.

The mechanical environment has significant effects on the maintenance, growth, and development of cartilage [8, 9], and similarly, the mechanical state of repaired articular cartilage are also regulated by cartilage repair and regeneration by tissue engineering in diarthrodial joints. The morphology, ultrastructure, and composition of articular cartilage results from the functional adaptations to its mechanical condition [1, 9, 10]. Cartilage tissue is sensitive to its mechanical environment [8–10]. A variety of excessive mechanical loading protocols result in both matrix damage and cell death [9, 10], while not enough mechanical loading exercise also causes cartilage damage [11]. Even moderate increases in load may be expected to accelerate the rates of both mechanical fatigue and wear [8]. When the conditions of repaired articular cartilage deviate from the physiological mechanical environment, cartilage degeneration may occur. Focal damage to articular cartilage is common in arthroscopy patients and may contribute to progressive tissue degeneration by altering the local mechanical environment [1, 12–14]. Under compression loading, stress concentration occurred in areas of cartilage near the edges of a focal defect, and local strains are increased markedly in these regions. Elevated strain may reach levels that induce cell death and matrix damage, and thus, the elevated strain resulting from a focal defect may become injurious to cartilage and induce degeneration [14]. Finite element models of joints with focal defects also predict the increased strain in the tissue adjacent to a defect [15].

Although the mechanical environment on focal defects of articular cartilage has been predicted theoretically and analyzed experimentally in in vitro systems, few reports focus on what the mechanical condition is after repairing cartilage defects by cartilage tissue engineering [10]. In current engineered cartilaginous tissue, most tissue-engineered constructs show inferior biomechanical properties compared with native articular cartilage in many patients [16, 17]. The biomechanical properties could cause local biomechanical environmental changes in the repaired region of the knee joint to damage the restoration similar to that of defects in articular cartilage. Finite element analysis showed that different strain phases occurred in the repaired region with a biphasic swelling model [18] and under rolling depression conditions. Some stresses in the tissue-engineered cartilage or host cartilage obviously deviated from normal physiological conditions of the knee [19, 20].

In this paper, we will establish both repaired models of full-thickness defects and half-thickness defects of articular cartilage. The mechanical characterization of a repaired articular cartilage region will be investigated in an experiment containing compressive load by digital image correlation technology.

## Methods

### Sample preparation

**Fig. 1:**
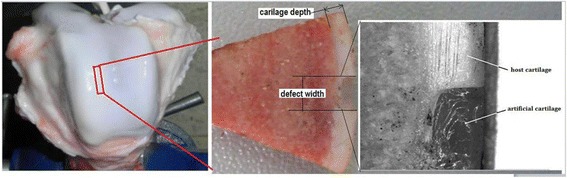
Repaired model of cartilage defect by using agarose gel

Eight-month-old pig knee joints were obtained from a local slaughterhouse. A fine saw was used to harvest a cartilage-bone block resembling a fan-shaped sheet (Fig. 1) by cutting along the perpendicular to the articular surface of the trochlea site. The full-thickness defect samples (*n* = 4) were made at the trochlea site by a curved knife, and the half-thickness defects samples (*n* = 4) also were obtained from the femoral groove. The cartilage defect sample is 4 ± 0.1 mm in length, and the depth of cartilage is 2 ± 0.14 mm. The dimension of the full-defect is 4 mm × 3 mm × 2.0 mm respectively corresponding to the samples’ length, defect width, and depth of cartilage. The dimensions of the half-thickness defect are 4 mm × 3 mm × 1.0 mm, also respectively corresponding to the samples’ length, defect width, depth of cartilage. Before conducting the experiment, both types of cartilage defect samples were kept moist in a culture dish with phosphate buffered saline (PBS).

The artificial cartilage was made of agarose gel at 1.8% concentration. Using the curved knife, a block of agarose gel was processed into a block of artificial cartilage with the same dimensions of the cartilage defect within the cartilage-bone block. Since the defect was processed by the same curved knife, this ensured the same shape of defects in the same samples. The Young’s modulus of the agarose gel is 20 kPa within linear elastic 0–0.6 strain and 50 kPa in nonlinear elastic 0.6–0.8 strain, which we obtained by mechanical testing. This modulus of 20 kPa of the gel corresponds to the modulus of tissue-engineering cartilage which is not functionally cultured under mechanical conditions [21].

The cartilage defect was repaired similarly as in clinical practice. The artificial cartilage was transplanted into the cartilage defect sample with the defect interface glued by the medical biological glue named Compont Medical Adhesive (Beijing Suncon Medical Adhesive Co.Ltd) which has been used in clinical surgery. Figure 1 shows the repaired model of cartilage defect using the application of agarose gel.

### Compression experiment

**Fig. 2:**
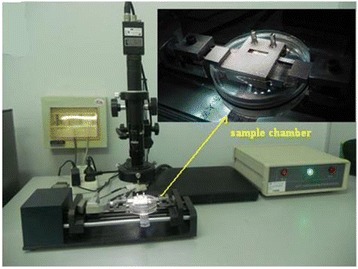
Testing machine and camera system as well as sample chamber

The compression tests of repaired cartilage defect sample were performed on a MTF-100 testing machine (MTF-100, ± 100 N, Center of Mechanical Experiment in Shanghai University, China) (Fig. 2). The speckle patterns on the repaired region were generated by iron oxide nanoparticles (Beijing Dk Nano technology Co., LTD). The nanoparticles are hard and black making them suitable for creating speckles and are embedded into the repaired tissue surface. This is so the clear identified images of cartilage can be captured by camera. The cartilage repaired region was compressed against a transparent glass in the sample chamber during the compression experiment to avoid off-plane displacement from the repaired region surface to ensure the accuracy of strain calculations. The sample chamber was filled with PBS at room temperature (Fig. 2).

The subchondral bone was gripped and had exerted on it a tiny preload by the testing machine. Prior to any compressive loading, the initial thickness of each sample was measured microscopically. An image of the sample in its load-free reference state was first acquired. The samples were compressed at a speed of 1 mm/min up to a 30% depth of cartilage at room temperature. During the loading process, samples are submerged in PBS, and interstitial fluid flowing from the compressed samples was dissolved into PBS, which ensures that clear images were captured by the camera. At the same time, the images of repaired cartilage were acquired at two frames per second during the entire testing process, and the images of the sample deformation were captured using the optimized DIC technique. The full-thickness defect samples and the half-thickness defects samples were loaded in the same conditions. The load was stopped when the apparent compression reached 30%. There was no cracking phenomenon at the interface of the repaired cartilage defect during the compression process.

### Strain testing location determination at feature site of repaired region

**Fig. 3:**
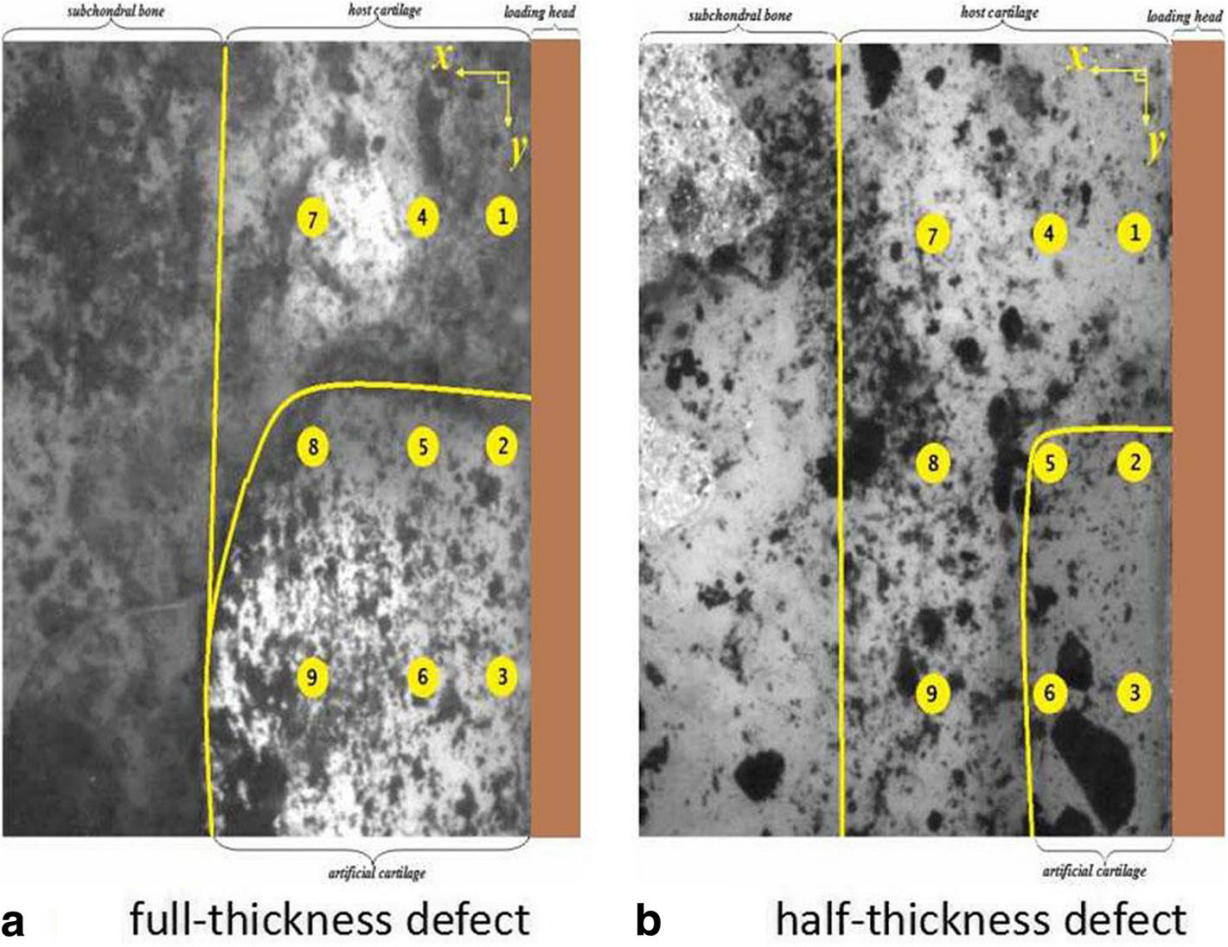
Cartilage defect samples. **a**: full-thickness defect; **b**: half-thickness defect. (Note: The yellow line shows the interface between artificial and host cartilage, and the right block part is a researched strain field). The location 1, 2, 3 represents the superficial zone of the host, interface and artificial tissue in repaired cartilage; The location 4, 5, 6 represents the middle zone of the host, interface and artificial tissue in repaired cartilage; The location 7, 8, 9 represents the deep zone of the host, interface and artificial tissue in repaired cartilage

Different locations of strain on the superficial, middle, and deep zones of the host, interface, and artificial tissue in repaired cartilage were researched. Locations of sub-regions are shown in Fig. 3: full-thickness defect sample including (1) representing the site of host cartilage (HC), (2) interface cartilage site (IC) between the host and artificial cartilage, and (3) artificial cartilage site (AC) in superficial zone(SD); (4) standing for HC, (5) IC, and (6) AC in middle zone(MZ); and (7) standing for HC, (8) IC, and (9) AC in deep zone(DZ). There are certain differences of location in half-thickness defect sample (Fig. 3). Location 6 is near the interface site in the bottom of the artificial cartilage. Location 8 in the half-defect sample is the host cartilage, while location 8 in the full-defect sample is in the artificial cartilage. Location 9 in the half-defect sample is the same in the host cartilage, while location 8 in the full-defect sample is same in the artificial cartilage.

The locations of sub-regions are arranged as follows: distance (D12) between location 1 and 2 = 1300μm; D23/D45/D56/D78/D89 = 1300μm; D14/D25/D36 = 550 μm; D47/D58/D69 = 850 μm. The locations 3, 6, and 9 are on the center line or symmetric line of the defect region from the surface to the depth of cartilage. Distance between location 1 and the surface of cartilage is 140 μm.

**Fig. 4:**
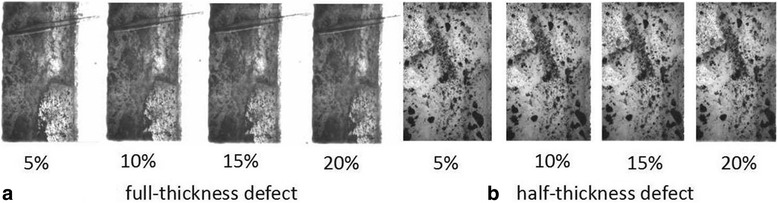
Serial images of full-thickness defect and half-thickness defect at different 5, 10, 15, and 20% compression

The testing location strain is calculated using the average value on sub-regions of the circle area with a radius of 10 pixels (~ 40 μm). The strain at the feature site of the repaired region was respectively calculated at cartilage compression of 5, 10, 15, and 20% (Fig. 4).

### Statistical analysis

For the tests, a one-way analysis of variance (ANOVA) was carried out to determine the statistical variances among the strain values of host, interface, and artificial cartilage zones and also in the superficial layer, middle, and deep zones of cartilage, respectively. A *p* value of < 0.05 was considered to be statistically significant. Test data used in the figures represented mean values, while the standard errors above and below mean values were indicated by error bars.

## Results

Overall, the strain profiles in both full-thickness and half-thickness defect models of cartilage exhibited heterogeneous distribution through the host cartilage (HC) to the interface(IC) and to artificial cartilage (AC).

### Full-thickness defects of cartilage models

**Fig. 5:**
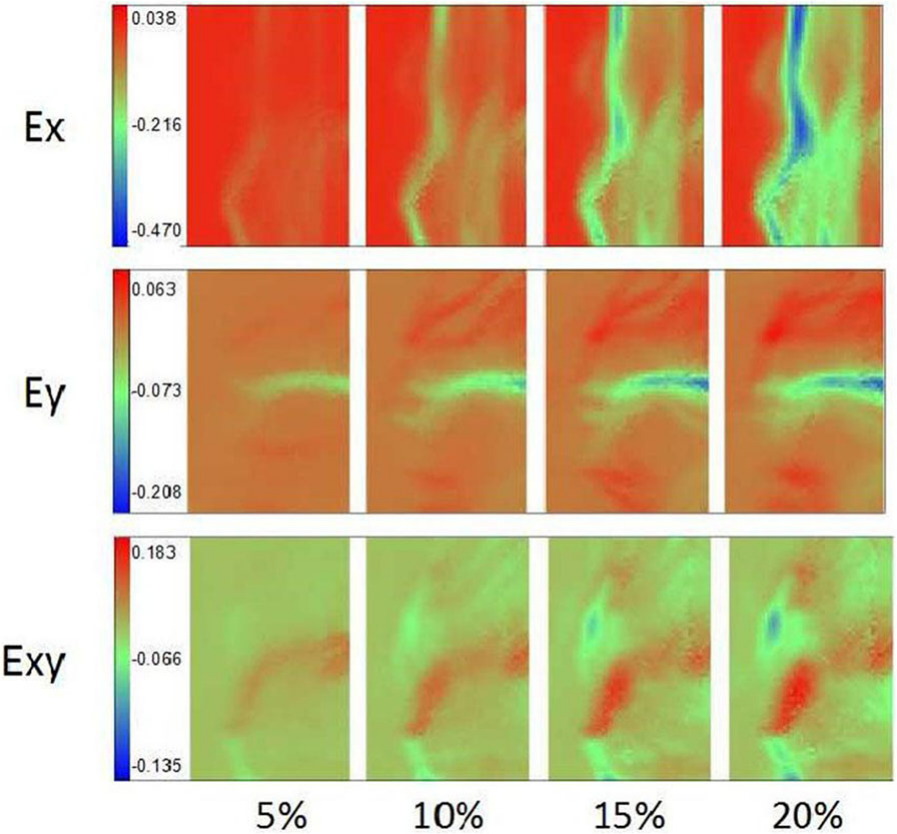
A reprensentative strain distribution about axial normal strain (E*x*) perpendicular to the surface of the cartilage and lateral normal strain (E*y*) as well as shear strain (E*xy*) change with increase of compression 5, 10, 15, and 20%

**Fig. 6:**
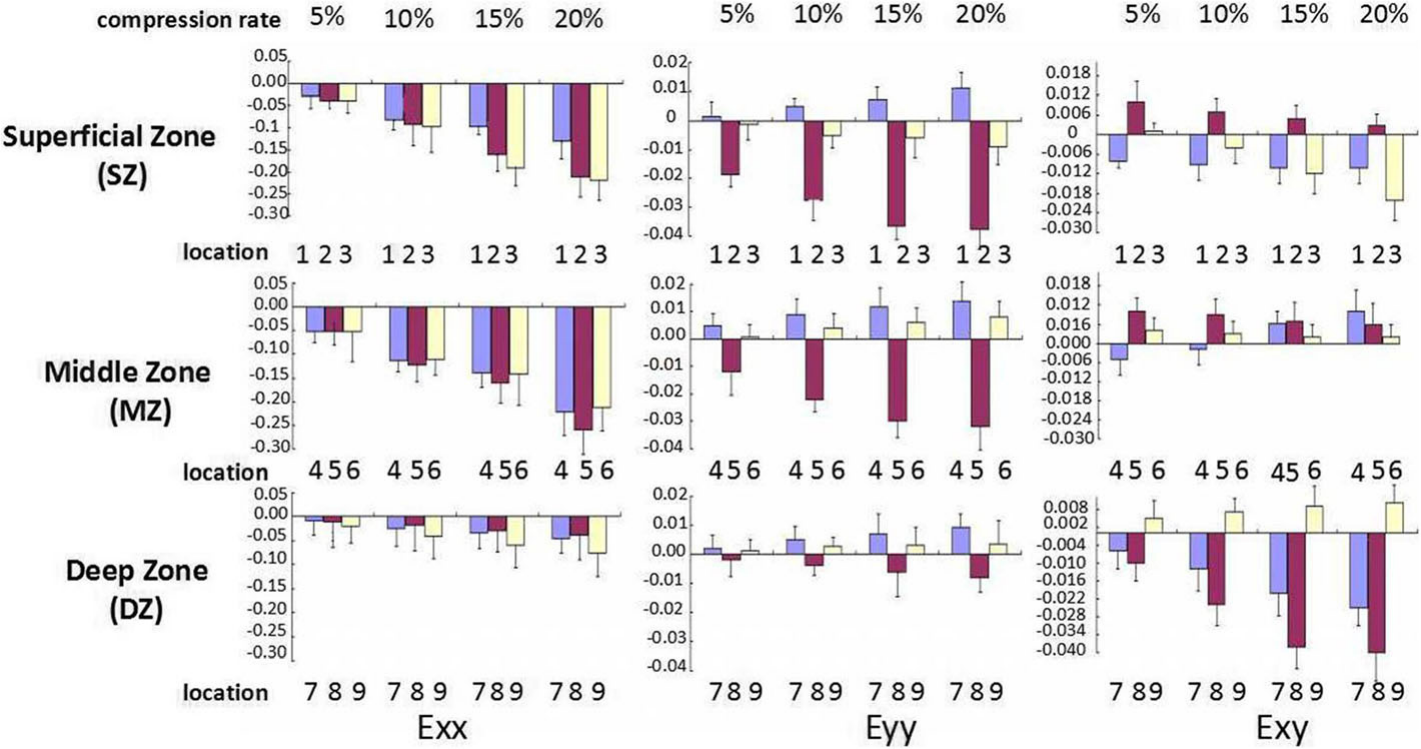
Different strains at different locations change with an increase of compression to 5, 10, 15, and 20% (location 1, 2, 3 standing for the host, interface, and artificial zone compared to repaired cartilage in superficial zone, 4, 5, 6 in middle and 7, 8, 9 in deep)

A representative strain distribution about axial normal strain (E*x*) and lateral normal strain (E*y*) as well as shear strain (E*xy*) with an increase of compression to 5, 10, 15, 20% was shown in Fig. 5. Different strains at locations 1, 2, 3 standing for the host, interface, and artificial zone compared to repaired cartilage in superficial zone, 4, 5, 6 in the middle and 7, 8, 9 in the deep zone were shown in Fig. 6.

In the X direction (perpendicular to the cartilage surface), the E*x* of the repaired zone presents compressive strain distribution with characteristics of depth-dependent profiles during compression. The compressive strains decreased with depth in the strain profile, and the E*x* increased gradually with loading increase, which is similar to other researchers have found [13]. The largest E*x* occurred at MZ about 1300 μm from cartilage surface. The E*x* distribution at AC is higher than that at HC in SZ and is also more uniform. The ∣E*x*∣of IC in DZ is larger than AC while lower than the one at HC in DZ during 5, 10, 15, 20% compression. However, there are no significant differences in E*x* among HC, IC, and AC (*p* = 0.644).

In the Y direction (parallel to the surface of cartilage), the E*y* of IC at SZ and MZ obviously occur compressive strain, and the ∣E*y*∣of SZ is larger than MD and DZ, whereas the Ey of HC and AC appears to be tensile strain, though the E*y* distribution is still depth-dependent like E*x* distribution. The ∣E*y*∣ of HC in SZ and MZ are larger than AC. The E*y* of IC at SZ increases rapidly at the start of compression and slowly at the end. At 20% compression, the maximum ∣E*y*∣of IC in SD is near four times as the one in AC, and 3.5 times as the value in HC. There is significantly different E*y* among HC, IC, and AC (*p* = 0.002).

As for the shear strain (E*xy*) in the restored region, the E*xy* within the repaired zone appears to display region-specific change at HC, IC, and AC. The E*xy* of IC within the repaired zone is significantly different from HC and AC (*p* = 0.001), even the E*xy* directions of AC in SZ and of HC in HC changes with increases in compression. The maximum∣E*xy*∣at IC of DZ is about four times the one at AC of DZ with repaired zone at 20% compression. The E*xy* of HC in DZ appears to go in the opposite direction of AC during the entire compression process.

### Half-thickness defects of cartilage models

**Fig. 7:**
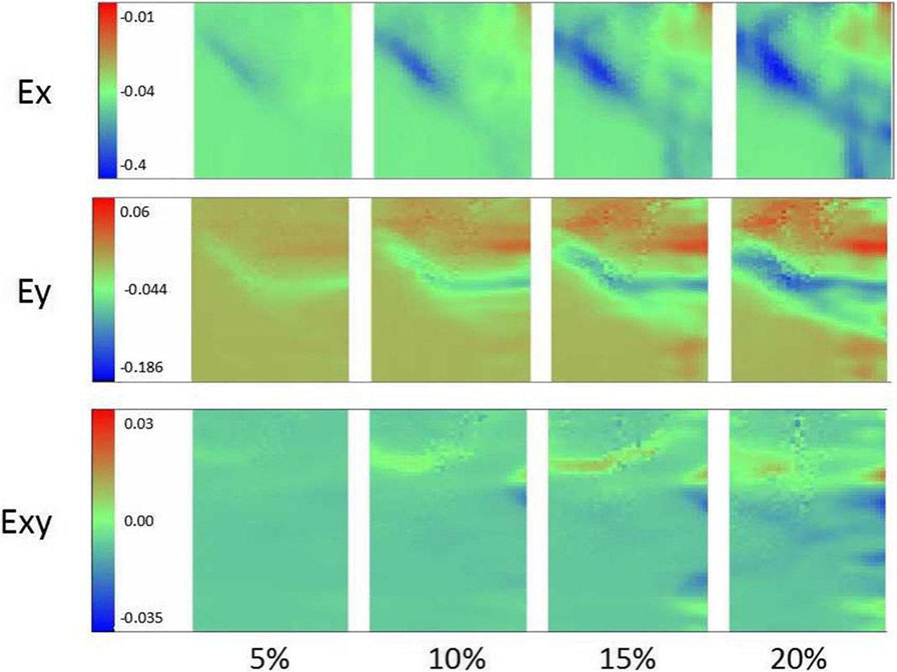
A representative strain distribution about axial normal strain (E*x*) perpendicular to the surface of the cartilage and lateral normal strain (E*y*) as well as shear strain(E*xy*) changes with increase of compression to 5, 10, 15, and 20%

**Fig. 8:**
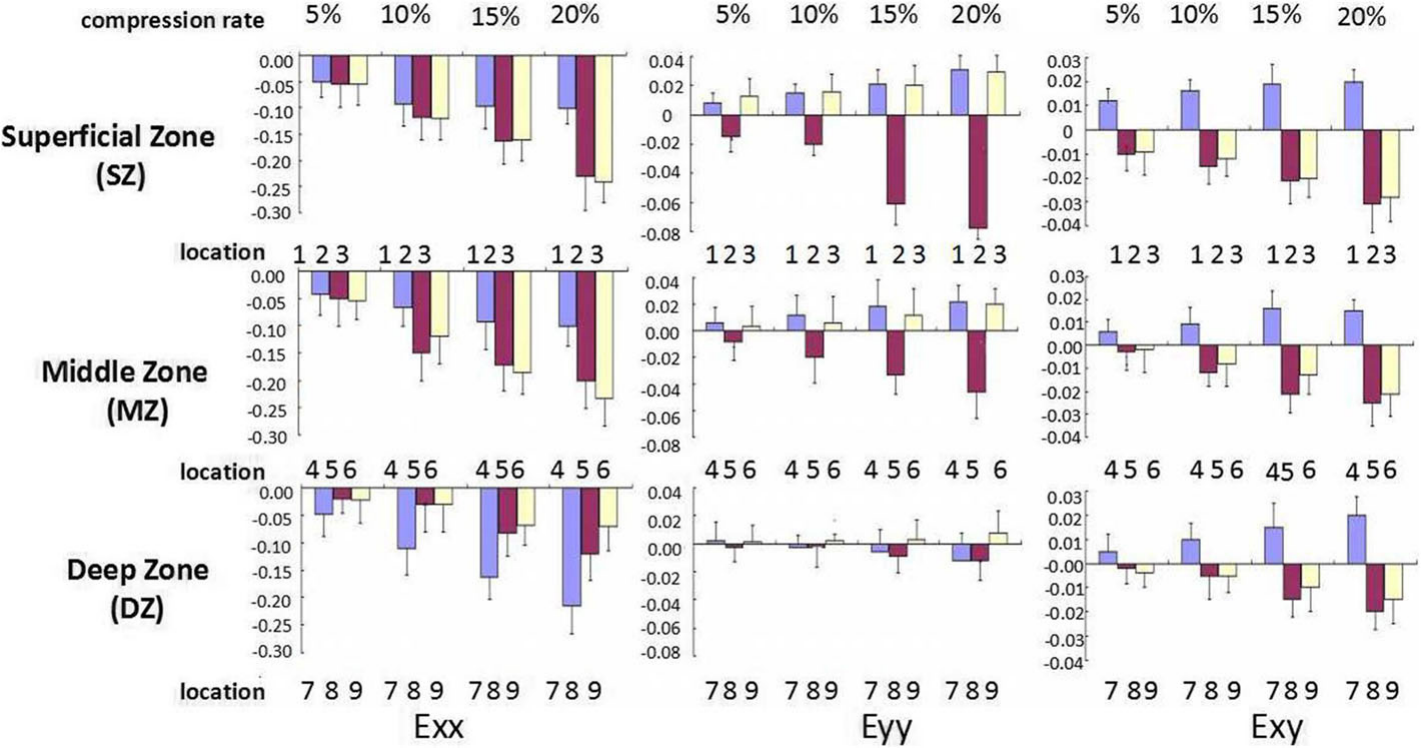
Different strains at different locations change with an increase of compression to 5, 10, 15, and 20% (location 1, 2, 3 standing for the host, interface, and artificial zone compared to repaired cartilage in superficial zone, 4, 5, 6 in middle and 7, 8, 9 in deep)

Like full-thickness defects, the half-thickness defects of cartilage have the same similar strain distribution at HC, IC, and AC except local differences. The strain distribution indeed exhibited region-specific changes within the repaired zone. A representative strain distribution about E*x*, E*y*, and E*xy* with increase of compression to 5, 10, 15, and 20% are shown in Fig. 7. Different strain at locations 1, 2, and 3 standing for the host, interface, and artificial zone compared to repaired cartilage in superficial zone, 4, 5, and 6 in the middle zone and 7, 8, and 9 in deep zones are shown in Fig. 8.

In the X direction (perpendicular to the cartilage surface), the E*x* distribution of AC is relatively more uniform than that of HC. At 20% compression, the maximum ∣E*x*∣ of HC is 3.2 times that in AC. There are significantly different E*x* among HC, IC, and AC (*p* = 0.002). In the Y direction, the E*y* of IC mainly exhibits the compressive strain distribution similar to that in the full-thickness defect, while the E*y* of HC and AC appear to have tensile strain at SD and MD. As for the XY direction shear strain, the direction and amount of E*xy* of HC is different from the one in IC and AC at the repaired region. These differences are obvious among the HC, IC, and AC (*p* = 0.002).

## Discussion

According to the clinical surgery method of cartilage tissue engineering, we researched the mechanical characteristics of a repaired region after articular cartilage defects were transplanted by artificial cartilage. The mechanical characteristics of the repaired region only represent the time just after implantation. Cartilage development is affected by many factors, such as biological, chemical, material, and biophysical. Here, the research only applies to biophysical force effects. In fact, a comprehensive study should be performed at different time periods, which will be our future goal.

The strain in the restored area of cartilage appears to have complex variation, and it is related to many factors in the restored area such as the interface bonding, the shape of the defect, material mechanical properties, and the loading condition. In this study, the defect interface was adhered by biological glue, which has been used in clinical surgery, in order to ensure the experiment operated in a realistic surgical environment. There was no cracking phenomenon at the defect interface during the apparent compression process. We will further research the cause of cracking at the defect interface being caused by incomplete bonding and stress concentration at the interface. For the shape of defect, the defect is classified as a full-thickness defect model and half-thickness defect model of cartilage, and both defect interfaces appear to possess obvious stress concentration. Moreover, the strain distribution in the restored area is also related to the size of artificial cartilage transplanted into the defect area. The different shapes of the defects are also lack of researches. The material mechanical properties of artificial cartilage play an important role in strain profiles in the repaired region. If the agarose gel is incompressible, implanted artificial cartilage will cause complex changes of E*x* and E*xy*. The strain in the restored area of cartilage indeed appears to have complex variation. The complex E*x* and E*xy* in this study may result from the artificial cartilage being incompressible. Compression as a loading force was applied in the experiment, and this kind of load is only typical of what is usually imposed on knee cartilage. In practice, articular cartilage is also affected by a rolling load and a sliding load, so it is essential to study the mechanical characteristics of the restored area of cartilage under these loads.

In the previous experimental cases, some scholars have established a finite element model of defect cartilage restoration to simulate and analyze the strain distribution at the restored area of host cartilage and artificial cartilage. This allowed them to explore the elastic modules, the amount of compression, and the size of defect area, and this affects the stress distribution at the restored area [20, 22]. The results show that the amount of compression obviously affects the strain distribution at the restored area. For example, when the elastic module of artificial cartilage is 0.6 MPa, the difference of strain between the artificial cartilage and host cartilage with compression loading ranging from 5 to 30% is up to 3.26 times. The size of the defect area is also related to the strain distribution of cartilage, and it is also consistent with the previous experimental conclusions. In this experiment, the variation trend of strain of host cartilage away from the restored area decreased gradually from superficial layer to deep layer. The strain field showed in this experiment is consistent with Ahmed, Gao, and Zhang’s findings who researched the strain distribution of intact cartilage respectively [23, 24].

In this experiment, a phenomenon was discovered in which the mechanical properties of artificial cartilage should be consistent with host cartilage, or otherwise, there will be residual stress after loading. When the compressive load is removed, the recovery timetable in the restored area of artificial cartilage and host cartilage appears to have obvious differences. The artificial cartilage recovered faster, whereas the host cartilage recovered slower because of its different viscoelastic properties. When the compression is at 30%, the host cartilage required 30 min to be restored to its original height. And that the defect interface appears to contain residual stress, and this will affect the cartilage restoration. This area of research will be looked at in the future.

In this study, the artificial cartilage will help to reveal one of the mechanical states in restoration of full-thickness and half-thickness defects of cartilage in only one kind of elastic modulus. At present, when the loading rate range is from 0.038 to 4.05% per second, the elastic modulus of artificial cartilage ranges from 0.94 to 3.035 Mpa [24]. The elastic modulus of agarose used in this experiment is about 20000pa, which is still lower than the native cartilage, but it is consistent with the mechanical properties of tissue-engineered cartilage at present. If the mechanical properties of the restored area need to be clearly researched after artificial cartilage is implanted into the defect cartilage, it is essential to use different concentrations of agarose to make a higher gradient elastic modulus to research. The agarose gel with an elastic modulus equal to or higher than cartilage is also needed to compare to each other. The carrageenan may be jointed to enhance the toughness and elastic modulus for researching the mechanical properties of the restored area of cartilage in the future.

## Conclusion

In the article, we simulated the artificial cartilage and host cartilage that is completely bonded, and is not completely bonded clinically, and its real boundary conditions are more complex, so that there are some differences between the simulation results and the actual situation. In addition, the agarose appears to have a higher brittleness in the experiment, which is different from the natural cartilage. On the other hand, pig knee joints and human knee have substantial differences in geometry and loads, but these experiments also have some theoretical and reference value; they reveal the mechanical behaviors of defect articular cartilage in a restored area with a compressive load. It will help to improve the mechanical properties of artificial cartilage implants and explore the suitable mechanical function indicators of artificial cartilage in implanting and restoration during cartilage tissue engineering in the future.

## Abbreviations

E*x*: Axial normal strain perpendicular to the surface of the cartilage
E*xy*: Shear strain
E*y*: Lateral normal strain
